# Comparison of the clinical features and outcomes of severe seasonal influenza and COVID‐19 patients in Tunisia between 2021 and 2022

**DOI:** 10.1111/irv.13215

**Published:** 2023-12-20

**Authors:** Jalila Ben Khelil, Rihab Yazidi, Nacef Ben Mrad, Fatma Jarraya, Emna Rachdi, Samia Ayed, Amira Jamoussi, Afif Ben Salah, Mohamed Besbes

**Affiliations:** ^1^ Intensive Care Department Abderrahman Mami Hospital Ariana Tunisia; ^2^ Faculty of Medicine of Tunis University of Tunis El Manar Tunis Tunisia; ^3^ Research Unit for Respiratory Failure and Mechanical Ventilation UR22SP01 Abderrahman Mami Hospital Ariana Tunisia; ^4^ Service of Medical Epidemiology Institut Pasteur de Tunis Tunis‐Belvédère Tunisia; ^5^ Laboratory of Transmission, Control and Immunobiology of Infections (LR16IPT02); Institut Pasteur de Tunis Tunis‐Belvédère Tunisia; ^6^ Department of Family and Community Medicine, College of Medicine and Medical Sciences (CMMS) Arabian Gulf University (AGU) Manama Bahrain

**Keywords:** comparison, COVID‐19, influenza, management, mortality, outcomes

## Abstract

**Background:**

We compared patients diagnosed at a SARI (severe acute respiratory infections) surveillance site with COVID‐19 and those with seasonal influenza to investigate the clinical differences, common features, and outcomes.

**Methods:**

We conducted a descriptive, retrospective study in the Medical Intensive Care Unit (ICU) at Abderrahman Mami Hospital between September 2021 and April 2022. Demographic, clinical, and biological data as well as outcomes were recorded for all patients.

**Results:**

Among 223 SARI patients, 83 were confirmed COVID‐19, and 22 were influenza positive. The distribution according to gender was similar; but patients with influenza were younger than those suffering from COVID‐19(mean age 60.36 SD 17.28 vs. 61.88 SD 17.91; P = 0.601). In terms of underlying chronic diseases, the frequency was 84.3% in the COVID‐19 group and 72.7% in the influenza group. COVID‐19 patients had a longer duration of hospitalization (mean [SD], 9.51 days [8.47 days] vs. 7.33 days [8.82 days]; P = 0.003), and a more frequent need for invasive ventilation (80 [97.4%] vs. 20 [92.3]). Case fatality was also higher among this group compared to the latter (39 [47%] vs. 6 [27.3%], P = 0.01).

**Conclusion:**

This exploratory study suggests higher severity of COVID‐19 compared to seasonal influenza among SARI hospitalized patients even during the Omicron wave. Further research on higher sample sizes is required to confirm this conclusion.

## 1 INTRODUCTION

Severe acute respiratory syndrome coronavirus‐2 (SARS‐CoV‐2) and influenza virus are significant causes of severe respiratory illnesses that can be fatal. Globally, as of December 8, 2022, a total of 642,924,560 confirmed cases of COVID‐19, including 6,625,029 deaths, have been reported to the World Health Organization (WHO).^1^ A 2018 study demonstrated that global influenza associated with respiratory mortality is occurring at a higher frequency than previously reported.^2^ As two respiratory viruses with pandemic potential, this raises concerns about how SARS‐CoV‐2 and the influenza viruses themselves, as well as public health measures and relevant scientific research, influence each other mutually. It is important to note also that these two respiratory diseases have many common characteristics that may interact in driving pandemics. The respiratory tract as a route of transmission allows these viruses to easily spread across space and over time. The considerable number of latent or asymptomatic infections and mild cases complicates the control of the disease.^3^ Several other common features to both viruses imply that knowledge can be shared in their control. Facing the complicated situation of co‐circulation of SARS‐CoV‐2 and influenza viruses with other respiratory pathogens, comprehensive management, prevention, and control strategies are needed. It is highly recommended to enhance the WHO's Global Influenza Surveillance and Response System to monitor the respiratory viruses. An effective continuous surveillance of any new variants is key also for providing early warnings. It is important for clinicians and epidemiologists to accurately identify these two respiratory infections via their differential clinical manifestations, especially because of their distinct treatments and prognoses. Furthermore, the lack of data surrounding comparison of the clinical features of influenza and COVID‐19 and the knowledge gaps in low‐income countries highlights the importance of conducting this study in Tunisia.

The aim of the present study was therefore to compare patients infected with COVID‐19 and those with seasonal influenza in order to investigate the clinical differences, common features, and outcomes.

## 2 METHODS

We conducted a retrospective study in the Medical Intensive Care Unit (ICU) at Abderrahman Mami Hospital in Ariana, Tunisia. With a capacity of 22 beds, this unit had an average number of intensive care admissions of approximately 550 per year. In 2015, the ICU of Abderrahman Mami Hospital was chosen as a sentinel center among six university hospital sites of various specialties to ensure the surveillance of severe acute respiratory infections (SARI) in Tunisia.^4^ This site participates in the surveillance of influenza from hospitalized cases as part of the National Influenza Surveillance and Control Program set up in Tunisia since 1980 within the Direction of Primary Health Care at Ministry of Health.^5^ In March 2020, it was selected as a reference center for COVID‐19 in Tunisia.

### 2.1 Study population and sampling

A total of 223 nasopharyngeal aspirates were obtained between September 2021 and April 2022from SARI patients admitted to the ICU at Abderrahman Mami Hospital. SARI cases were selected according to the WHO revised clinical case definition as all acute respiratory illness cases with a history of fever or measured fever of ≥38°C and cough, with onset within the past 10 days, requiring hospitalization.^6^ Once collected, samples were submitted to the Laboratory of Microbiology at Abderrahmane Mami hospital and to the Laboratory of Virology at Charles Nicolle Hospital for bacteriological and virologic diagnosis, respectively. Virologic aliquots were transported in viral transport medium (Universal Transport Medium; COPAN Diagnostics Inc., Murrieta, CA) and at a temperature of 4°C to the laboratory. For each sample, demographic and clinical data were collected. The report form included questions on gender, age, general demographic information, clinical signs, and health status. Bacterial diagnosis included microscopy direct examination and culture according to conventional methods. The virology diagnosis included molecular detection of influenza A and B viruses, parainfluenza viruses, respiratory syncytial virus (RSV), human metapneumovirus, adenovirus, coronavirus, bocavirus, and *Enterovirus* genus (HRV and HEV) using a multiplex nucleic acid amplification panel (xTAG RVP FAST v2, Luminex Molecular Diagnostics, Austin, TX, USA). Rapid direct RSV antigen detection was also performed using Alere BinaxNOW® RSV Card (Abbott Diagnostics).

### 2.2 Statistical analysis

Descriptive statistics of the patients were performed and reported in terms of absolute frequencies and percentages for the qualitative variables. The association between type of infecting pathogens and clinical manifestations/outcomes were performed using the chi‐square (χ2) test or Fisher's exact test (where cell counts below 5 were encountered in the statistical table). Variables with a P value <0.2 in the univariate analysis were entered into multivariate logistic regression analysis to assess statistical associations between independent variables and COVID‐19 or influenza. All P values <0.05 are considered statistically significant. A value of P ≤ 0.05 was considered as significant. SPSS 19.0 was used for statistical analyses.

## 3 RESULTS

From September 1, 2021, to April 30, 2022, there were a total of 223 SARI patients SARI patients admitted to the ICU at Abderrahman Mami Hospital, among which 83 were confirmed COVID‐19 and 22 were influenza positive, all of which are confirmed Influenza A (H3N2). For the remaining 118 patients, other pathogens were identified as adenovirus (n = 4), respiratory syncytial virus (n = 3), Human bocavirus (n = 3), metapneumovirus (n = 2), enterovirus (n = 2), rhinovirus (n = 15), and parainfluenza (1, 2, 3, and 4) (n = 4).

### 3.1 COVID‐19 and influenza patient characteristics

The mean age of patients with COVID‐19 was 60.36 (SD 17.28) years, which was lower than that of patients with influenza (61.88 SD 17.91). There was no significant difference according to gender in the COVID‐19 and influenza groups (54.5% vs. 54.9%, respectively; P = NS). For both groups, more than 60% of admitted patients are over 60 years old, and more than a third are smokers (COVID‐19 [38.6], influenza [35.3]). In terms of underlying diseases, 84.3% (70) of patients with COVID‐19 have at list one comorbidity versus 72.72% (16) of influenza patients (P = 0.045). The most frequent comorbid conditions in both groups were cardiovascular diseases and diabetes. However, there were a significantly greater number of patients with asthma and respiratory chronic diseases in the influenza group. More than 30 % COVID‐19 patients were obese (Table 1). There is no significant difference in functional symptomatology (cough, fever, and onset within the past 10 days) between COVID‐19 and influenza patients.

**TABLE 1:** Baseline and admission characteristics of patients admitted to intensive care unit with seasonal influenza and coronavirus disease 2019 (COVID‐19).

Characteristics	Overall (N = 223)	COVID‐19 (n = 83)	Seasonal influenza (n = 22)	OR (95% CI)	P value*	AOR (95% CI)	P value**
Mean (SD) age, years	58.95 (17.89)	60.36 (17.28)	61.88 (17.91)	—	—		
Sex							
Male	55.2 (123)	55.4 (46)	54.5 (12)	1.01 (0.59–1.72)	0.959		
Female	44.8 (100)	44.6 (37)	45.5 (10)	1			
Age category, years							
≤18	2.2 (5)	1.2 (1)	4.5 (1)	1.76 (0.99–1.37)	0.064		
18–29	6.7 (15)	6.0 (5)	0.0 (0)				
30–39	8.5 (19)	7.2 (6)	13.6 (3)				
40–49	6.7 (15)	9.6 (8)	4.5 (1)				
50–59	16.6 (37)	12.0 (10)	9.1 (2)				
60–69	29.6 (66)	25.3 (21)	40.9 (9)				
≥70	28.3 (63)	38.6 (32)	27.3 (6)				
Smoking	39.0 (87)	38.6 (32)	35.3 (6)	1.51 (0.86–2.66)	0.148		
Comorbidities							
Cardiovascular diseases	37.7 (84)	51.8 (43)	40.9 (9)	2.55 (1.25–5.17)	0.009	2.71(1.11–6.61)	0.027
Neurologic diseases	8.1 (18)	6 (7.2)	9.1 (2)	1.87 (0.66–5.25)	0.235		
Diabetes	26.5 (59)	30.1 (25)	31.8 (7)	1.38 (0.69–2.76)	0.357		
Asthma	18.4 (41)	13.3 (11)	18.2 (4)	2.15 (0.99–4.64)	0.050		
Respiratory chronic diseases	30.0 (67)	24.1 (20)	50.0 (11)	1.64 (0.83–3.23)	0.147		
Obese (≥30)	27.1 (23)	31.7 (13)	2.4 (2)	1.16 (0.75–1.81)	0.489		

*Note*: Values are percentages (numbers) unless stated otherwise.

Abbreviations: AOR, adjusted odds ratio; CI, confidence Interval; OR, odds ratio.

* P < 0.2 (predetermined statistical significance level).

** P < 0.05 (predetermined statistical significance level).

### 3.2 Comparative clinical epidemiology and outcomes of COVID‐19 and influenza

**FIGURE 1:**
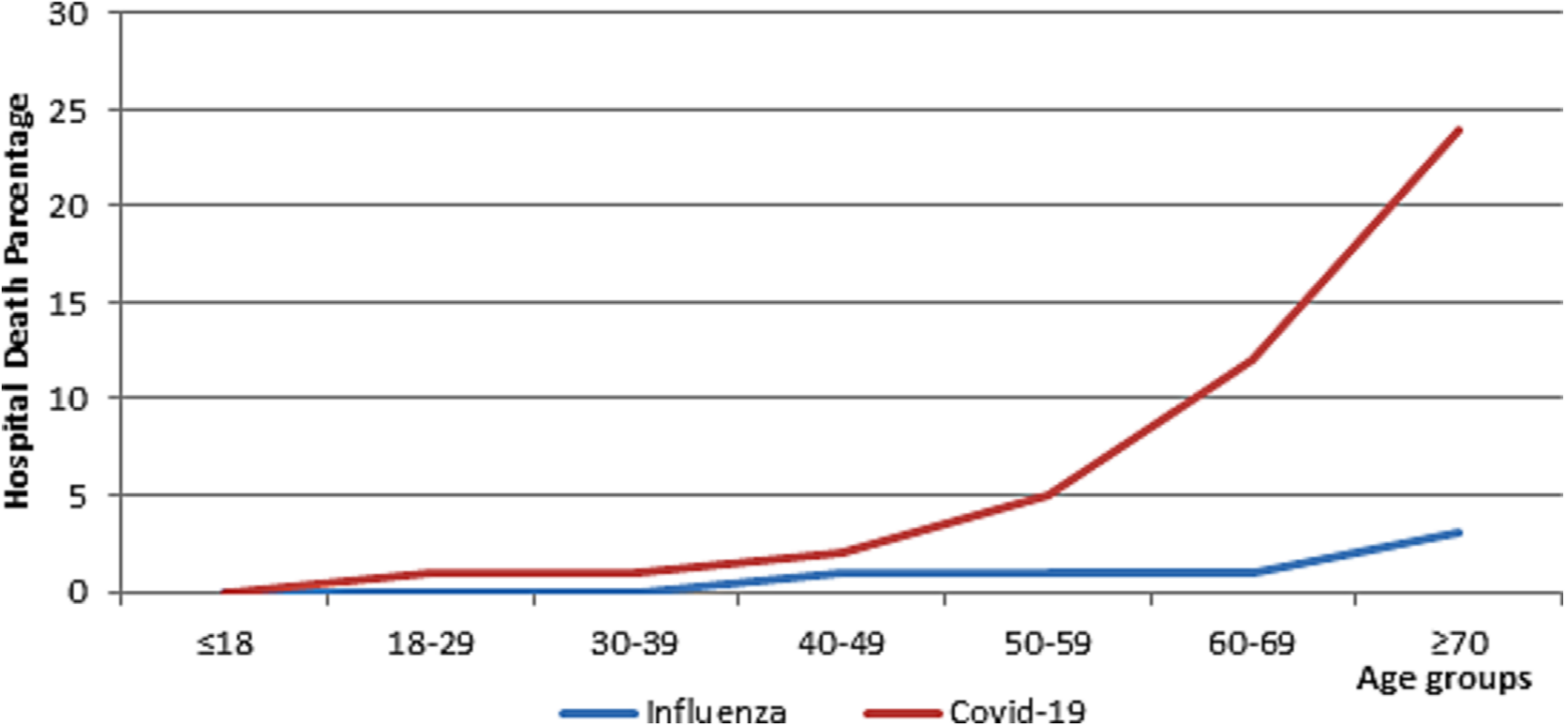
Percentage of deaths in patients hospitalized with influenza compared with patients hospitalized with COVID‐19 by age. *The age groups are divided by years.

Patients hospitalized with COVID‐19 had substantially worse outcomes than patients hospitalized with influenza. Over 89 % of patients with COVID‐19 required mechanical ventilation versus 54.5% with influenza (P = 0.011). In addition, patients with COVID‐19 were more likely to be treated with systemic steroids than patients with influenza (68.7% vs. 13.6%, P < 0.01). Besides, 38.1% of patients with COVID‐19 received concurrent antibiotics compared to 13.6% of patients with influenza (P = 0.033). COVID‐19 patients had a longer duration of hospitalization than patients with influenza (9.51 days [8.47 days] vs. 7.33 days [8.82 days]; P = 0.003) and a more frequent need for mechanical ventilation (80 [97.4%] vs. 20 [92.3]). The overall hospital mortality was most important in COVID‐19 patients compared with influenza patients (47.0% [39] vs. 27.3% [6]; P = 0.01) (Table 2), and the higher inpatient death rate was most prominent with increasing age (Figure 1).

**TABLE 2:** Hospital‐related outcomes for patients with COVID‐19 compared to patients with influenza.

	Overall (N = 223)	COVID‐19 (n = 83)	Seasonal influenza (n = 22)	P value
Management				
Systemic steroids	38.6 (86)	68.7 (57)	13.6 (3)	0.000
Antibiotics	38.1 (85)	38.5 (32)	13.6 (3)	0.033
Mechanical ventilation	74.9 (176)	97.4 (80)	92.3 (20)	0.607
Outcomes				
Hospital length of stay (mean; SD), days	9.3 (11.07)	9.51 (8.47)	7.33 (8.82)	0.003
Died during hospitalization	30.5 (68)	47.0 (39)	27.3 (6)	0.01

*Note*: Values are percentages (numbers) unless stated otherwise.

Abbreviation: SD, standard deviation.

In the multivariate model, cardiovascular diseases (AOR = 2.71[1.11–6.61]; P = 0.027) was the only factor independently associated with COVID‐19 in SARI cases.

## 4 DISCUSSION

To our knowledge, this study is the first, in Tunisia, comparing clinical features of SARI cases caused by COVID‐19 and seasonal influenza. This comparative approach allowed us to identify the common characteristics and the differences between these two respiratory infections in the same hospital setting and to provide indications for a better management of hospital resources and capacities. It confirmed that COVID‐19 is more severe even during the Omicron variant for cases admitted in intensive care units. In our study, there was no significant difference between the mean ages of patients with COVID‐19 and with influenza, and more than 60% of admitted patients for both groups were over 60 years old. The same results were reported by Talbot et al.^7^ Indeed, in this study,^7^ overall, patients with COVID‐19 were similar in age to those with influenza (COVID‐19: mean 60 years, median 61 years vs. influenza: mean 63 years, median 64 years). On the other hand, patients with SARS‐CoV‐2 infection were younger (median age [IQR], 59 [45–71] vs. 66 [52–77]; P < 0.001) in the study of Brehm et al.^8^, ^9^, ^10^ In this same study,^8^ the authors reported a higher proportion of men among COVID‐19 patients than in influenza patients (111 [66.9%] vs. 144 [56.5%]; P = 0.03) contrasting with our finding showing no significant difference between the proportions of male subjects in the COVID‐19 and influenza groups (54.5% vs. 54.9%, respectively). On the other hand, some previous studies demonstrated a higher overall incidence of seasonal influenza and higher disease severity in male compared to female patients.^11^, ^12^ Our analyses found that hospitalized COVID‐19 patients had higher underlying conditions, increased need for mechanical ventilation, and much rates of death than those hospitalized for seasonal influenza. Among the differences observed in this study was a higher frequency of death following hospitalization in patients with COVID‐19,^13^, ^14^ contrary to other studies which find no difference in mortality in patients who required hospitalization for COVID‐19 and influenza.^15^, ^16^ Patients hospitalized with COVID‐19 were more likely to have a pre‐existing comorbid condition compared with patients hospitalized with influenza (84.3% vs. 72.72%; P = 0.045). This result contradicts that of Talbot et al with a lower proportion of comorbidities in patients hospitalized for COVID‐19.^7^ Diabetes and cardiovascular diseases were the most frequent comorbidities in both groups of our patients; however, respiratory chronic diseases were a significantly more frequent in the influenza group. In the literature, COVID‐19 patients also suffered significantly less frequently from arterial hypertension, cardiovascular disease, chronic respiratory disease, chronic liver disease, and chronic renal disease and were less frequently solid organ transplant recipients compared to patients with influenza.^7^, ^8^ Because of the use of SARI cases definition recommended by the WHO,^6^ there were no differences between the two groups in term of clinical manifestations at admission in our study. Few studies reported some common symptoms in COVID and influenza‐like fever/feverish (77%), shortness of breath (80%), and fatigue (48%).^16^, ^17^ Among the differences observed in this study were increased need for mechanical ventilation, higher length of stay, and much higher rates of death in patients hospitalized for COVID‐19 compared to those hospitalized for seasonal influenza. These differences may be explained by preexisting influenza immunity and influenza vaccination. Although the H3N2 subtype, described as having the greatest impact on increased morbidity and estimated mortality,^18^, ^19^ accounted for all of our inpatients, mortality was significantly higher in COVID‐19 patients. A higher frequency of death following hospitalization in patients with COVID‐19 was reported by many studies^13^, ^14^; this result is controversial in another study.^15^ For COVID‐19, age is defined factor of death, and COVID‐19 patients were more likely to have died during hospitalization than influenza patients.^20^ COVID‐19 patients have a much longer length of stay than has been observed in patients with influenza.

This finding could be verified if we better characterize morbidity profiles of hospitalized severe cases of influenza compared to SARS‐CoV‐2 SARI cases. Indeed, this finding could simply reflect a classification or an under‐reporting bias of the comorbidities in the groups. We noticed the high prescriptions of more aggressive treatments and procedures among COVID‐19 SARI admitted cases as well as a higher case fatality. Unexpectedly, the sharp increase of the death rate with age was also noticed among this group. These findings could be helpful in the management plans for SARI patients that will be admitted in the new influenza season where both agents are still co‐circulating.

### 4.1 Limitations of the study

Despite the importance of findings provided by this innovative study in Tunisia, the conclusions need to be supported by larger sample size to detect accurately the differences of clinical features, management plans, and outcomes. A prospective study with high subgroup samples or a matched case–control study with powered sample size could help controlling for confounders by the study design. Knowing the differences between COVID‐19 and influenza symptoms seems essential and may help clinicians in the diagnosis of these diseases.

## 5 CONCLUSIONS

This study is the first throwing some lights about the clinical features and outcomes of COVID‐19 and seasonal influenza SARI cases admitted in Tunisia. Conclusions must be taken with care because of the small sample size and the preliminary character of the study. More robust study designs implemented in multiple centers could provide higher validity findings to guide future management plans.

These clinical differences can help the clinicians in front of cases with influenza like illness during the co‐circulation of influenza and SARS‐CoV‐2.

## AUTHOR CONTRIBUTIONS

**Jalila Ben Khelil:** Conceptualization; data curation; formal analysis; funding acquisition; investigation; methodology; project administration; software; supervision; validation; visualization; writing—original draft; writing—review and editing. **Rihab Yazidi:** Data curation; formal analysis; investigation; methodology; software; validation; writing—original draft; writing—review and editing. **Nacef Ben Mrad:** Data curation; investigation; project administration. **Fatma Jarraya:** Data curation; investigation; project administration. **Emna Rachdi:** Data curation; investigation; project administration. **Samia Ayed:** Data curation; formal analysis; investigation; methodology; supervision; validation; visualization. **Amira Jamoussi:** Conceptualization; investigation; methodology; project administration; visualization. **Afif Ben Salah:** Methodology; project administration; validation; writing—original draft; writing—review and editing. **Mohamed Besbes:** Conceptualization; funding acquisition; investigation; methodology; project administration; supervision; validation; visualization.

## CONFLICT OF INTEREST STATEMENT

The authors declare that they have no competing interests.

## ETHICS STATEMENT

The Tunisian Ministry of Health consider that sentinel surveillance for influenza is a part of routine public health surveillance and therefore did not require formal ethical review because data reported are used for public health surveillance purposes. Data were anonymized upon collection, and authors did not have access to identifying information.

### PEER REVIEW

The peer review history for this article is available at https://www.webofscience.com/api/gateway/wos/peer-review/10.1111/irv.13215.

## Data Availability

Data are available on request due to privacy/ethical restrictions.
